# Suicide risk factors among Lithuanian medical doctors and residents

**DOI:** 10.1186/s13690-024-01478-z

**Published:** 2024-12-24

**Authors:** Povilas Kavaliauskas, Evaldas Kazlauskas, Giedre Smailyte

**Affiliations:** 1 Department of Public Health, Institute of Health Sciences, Faculty of Medicine, Vilnius University, M. K. Čiurlionio Str. 21/27, Vilnius, LT-03101 Lithuania; 2 Laboratory of Cancer Epidemiology, National Cancer Institute, Vilnius, Lithuania; 3 Center for Psychotraumatology, Institute of Psychology, Vilnius University, Vilnius, Lithuania

**Keywords:** Medical doctors and residents, Lithuania, Suicidal behavior, Suicide risk

## Abstract

**Background:**

Medical doctors and residents are regularly exposed to multiple stressors that may lead to mental health problems. Work-related stressors contribute to elevated levels of psychological distress, anxiety, and depression among health care workers. This is the first investigation evaluating suicidal behaviour and thoughts among Lithuanian medical doctors and residents exposed to various professional stressors at two years after the start of the COVID-19 pandemic. The aim of the study was to evaluate suicidality and factors associated with high suicide risk in a large sample of Lithuanian medical doctors and residents.

**Methods:**

The research included 685 participants who completed an online questionnaire over a two-month period in December 2021 and January 2022. Medical doctors and residents from all specialties were invited to participate in the survey. The most common stressors in their work environment were measured. Mental health was assessed using the Depression, Anxiety and Stress Scale-21 (DASS-21) scale, and suicidality was measured with the Suicidal Behaviors Questionnaire-Revised (SBQ-R).

**Results:**

The lifetime suicide risk was found in 30.4% of the sample. Moreover, 11.4% of medical doctors and residents were identified as having previous or current suicide planning ideation, and 2.5% reported a previous suicide attempt. Univariate analysis showed that younger age, having no long-term relationships, shorter work experience, career change ideation, higher depression and anxiety, poor working conditions, at the direct contact with patients, lack of career perspectives, and exposure to mobbing and exhaustion at work were statistically significant risk factors for higher suicidality. Furthermore, regression analysis supported that having no long-term relationship, high depression, and high anxiety were significant risk factors for suicide risk in the sample.

**Conclusion:**

We found out that almost one-third of medical doctors and residents had lifetime suicide ideations and behaviours at the high suicide risk level. Main suicide risk factors were poor mental health, work-related stressors, and a lack of long-term relationships. The results of the study can help to develop prevention strategies by identifying populations that may be at high risk for mental health problems and provide evidence in implementing specific interventions to address mental health problems in healthcare workers.

**Table:** 

Text box 1. Contributions to the literature
• Medical doctors encounter enormous demands, high workloads, and long working hours. Work-related exhaustion may negatively affect mental health, such as depression, anxiety problems, and suicidal thoughts.
• Our data offers profound insights into the mental health of Lithuanian medical doctors. Given the dearth of studies on occupation-related mental health issues in our country, this study establishes baseline data for future investigations.
• Implementing mental health policies, prevention programs, and our data-based interventions in daily practice is mandatory.

## Background

Healthcare workers are routinely exposed to professional stressors that may increase mental health problems, including a higher risk for suicidal behaviour [1, 2]. Previous studies have indicated that physicians are at higher risk of suicide than the general population [3]. A meta-analysis by Dutheil et al. [4] concluded that physicians are professionals at risk for a suicide attempt, and female gender is a risk factor in this professional group. A study from Aasland analysing 40 years of mortality rates of Norwegian doctors showed that doctors had higher mortality rates than other professions representatives due to higher suicide rates among doctors [5]. Historically, physician suicide rates were 1.5 times higher than in the general population. However, the gap between the general population and physician suicide rates is closing [6]. Similar results are seen among medical doctors in Denmark’s population, where total suicide rates were 1.6 times higher than the general population [7].

The COVID-19 pandemic increased psychological distress, anxiety, and depression among healthcare workers [8, 9]. Studies conducted during COVID-19 reported different levels of suicidal ideation among doctors and other healthcare professionals. A study from the United Kingdom found that in six COVID-19 pandemic months, suicidal ideation among healthcare workers slightly increased from 10.8 to 11.3% of respondents [10]. A review from Groves et al. [11] showed that among nursing professionals, psychiatric, psychological, physical, occupational, and alcohol problems contributed to the higher suicide risk during the COVID-19 pandemic. In addition, research in Mexico found that 62% of nurses and 52.7% of doctors had suicidal risk [12].

This study aimed to evaluate suicidality and factors associated with suicide risk among medical doctors and residents in Lithuania at the two years after the start of COVID-19. In particular, we aimed to explore suicidality, identify the prevalence of suicide risk among medical doctors and residents, and identify how anxiety, depression, and various professional stressors are associated with high suicide risk.

## Methods

### Participants and procedure

The data collection period lasted from December 2021 to January 2022. We invited participants to respond to an online survey during this two-month period. Invitations to participate in the study were distributed through professional unions and associations of medical doctors, internal hospital networks, and Lithuanian doctors and residents social network groups. Participants were reminded twice to complete questionnaires after the initial invitation. Data from a total of 561 medical doctors and 124 residents who completed the survey were included in the analysis.

### Sociodemographic statistics and covariates

General demographic data was collected, including gender, age, relationship status, profession, type of work, level of medical service provision, size of the city, where they work, workload, and work experience after finishing training. A comprehensive search of the available literature was carried out with the purpose of identifying factors that are associated with poorer mental health among medical doctors. For the purpose of evaluating the role of factors that affect mood disorders, stress, and burnout, a comprehensive list of negative factors that have an impact on mental health was extracted from a high-volume meta-analysis [1, 2, 13–16]. These factors included poor working conditions, high workload, work with patients, lack of professional development, lack of career perspectives, problems with managers, mobbing, and exhaustion. Respondents were asked to indicate if any of those factors affected their daily lives by responding on a binary “yes/no” to the items listing these factors.

### Suicide risk and psychological distress

The Suicidal Behaviors Questionnaire-Revised (SBQ-R) was used to evaluate suicidality in the sample [17]. The American Psychological Association describes suicidality as the risk of suicide, usually indicated by suicidal ideation or intent [18]. The SBQ-R comprises four items, each covering a different dimension of suicidality: the first item - lifetime suicidal ideation and attempts; the second - frequency of suicidal ideation over the preceding 12 months; the third - the threat of suicide attempts; and lastly, the fourth - self-reported probability of suicidal behaviour in the future. The four SBQ-R items are each rated on Likert type scale. The total score of the SBQ-R is the sum of responses to all four items and range between 3 and 18 with higher score indicating a higher suicidality and a larger suicide risk. A sum of points with a cut-off of ≥ 7 indicates high suicide risk for the general population studies. The Cronbach alpha for this scale was 0.8. The four SBQ-R items allow for the collection of a wide variety of information. The cut-off score of the SBQ-R may be used for screening for the suicide risk in the sample, and individual items tap into various aspects of suicidality, e.g., Item 1: lifetime suicide ideation and/or suicide attempts.

The Depression, Anxiety and Stress Scale − 21 (DASS-21) was used for the assessment of depression and anxiety [19]. The DASS-21 is a widely used self-report measure that includes three subscales, measuring depression, anxiety, and stress levels. Each subscale consists of seven items measured on a 4-point Likert scale ranging from 0 (did not apply to me at all) to 3 (applied to me most of the time). The DASS-21 subscales are scored by summing the responses to each of the item with a higher score indicating higher depression or anxiety. We used these cut-off scores to identify the severity of depression and anxiety based on the previous studies: depression (normal < 9; mild 10–13; moderate 14–20; severe 21–27; extremely severe ≥ 28), anxiety (normal < 7; mild 8–9; moderate 10–14; severe 15–19; extremely severe ≥ 20). In this study, only two scales (DASS-21) were included in the data analysis. The Cronbach alpha for depression and anxiety scales were 0.89 and 0.82, respectively.

### Data analysis

Statistical analysis was performed using IBM SPSS 26.0. Chi-square and Student-t tests were used for univariate analysis to identify statistically significant risk factors for suicidal ideation. Hierarchical binary logistic regression was performed to assess predictors of high suicide risk in a multivariate analysis. Results were held statistically significant when *p* < 0.05.

## Results

The sample included 685 medical doctors and residents. The age ranged from 22 to 76 years. Most participants were female (78.7%) and in a long-term relationship (81.2%). Detailed sociodemographic statistics are shown in Table 1. A total of 208 (30.4%) respondents scored ≥ 7 on the SBQ-R, indicating lifetime suicide risk. Based on the SBQ-R first item, which measures suicide ideation, 282 (41.2%) of respondents reported they had suicidal thoughts in their lifetime. Furthermore, previous suicidal planning was reported by 78 (11.4%) of respondents, and 17 (2.5%) respondents reported previous suicide attempts, of whom 11 attempted suicide once, 5 twice, and 1 three times.

Work-related stress factors associated with suicidal behaviour were high workload, lack of professional development, lack of career perspectives, mobbing, and exhaustion. Detailed analysis is represented in Table 2.

Hierarchical binary logistic regression was performed to assess the role of factors associated with high suicide risk in the sample. The first model contained three independent variables (age and gender). The complete model was statistically significant, χ^2^ (2) = 12.93, *p* = 0.002, indicating that the model was able to distinguish between low-risk and high suicide risk participants. The model explained between 1.9% (Cox and Snell R square) and 2.6% (Nagelkerke R squared) of the variance in suicide risk and correctly classified 69.6% of cases. One significant predictor of reporting high suicide risk was age, with an odds ratio of 0.981.

In the second model, which was statistically significant, χ^2^ (6) = 57.48 *p* < 0.001, we added three statistically significant, not adjustable factors: relationship status, having children, and career change ideation. The model improved and explained between 9.7% (Cox and Snell R square) and 13.9% (Nagelkerke R squared) of the variance in suicide risk status and correctly classified 72% of cases.

In the third model, significant work-related stress factors were added to the model: poor working conditions, direct contact with patients, lack of career perspectives, mobbing, and exhaustion. The entire model containing all predictors was statistically significant, χ^2^ (11) = 68.15, *p* < 0.001. The model explained between 11.4% (Cox and Snell R square) and 16.3% (Nagelkerke R squared) of the variance in suicidal risk and correctly classified 71.5% of cases.

The fourth and final model in Table 3 included mental health indicators – depression and anxiety as predictors. The entire model containing all predictors was statistically significant, χ^2^ (12) = 130,87, *p* < 0.001. The model explained between 20.8% (Cox and Snell R square) and 29.7% (Nagelkerke R squared) of the variance in suicide risk and correctly classified 77.4% of cases. After adding mental health factors, three statistically significant predictors for high suicide risk were left, which were depression, anxiety, and not being in a long-term relationship. Detailed analysis is reported in Table 3.

## Discussion

We present the first study of Lithuanian medical doctors’ and residents’ suicidality in the late COVID-19 pandemic period. We found out that almost one-third of respondents had lifetime suicide ideation and behaviors at the high suicide risk level, and the most significant factors associated with the high suicide risk in our study were depression, various work-related stressors, and having no long-term relationship.

First, we identified that 30.4% of medical doctors and residents had suicide risk. Study findings are comparable to other studies that explored suicide risk in medical students and medical doctors; however, we found higher levels of suicidality in comparison to other studies. A study from India analysing medical students found even higher results: 37.2% of respondents had suicidal ideation, 10.9% planned suicide, and 3.3% mentioned attempting suicide [20]. Among German veterinary students, suicidal ideation reached 19.9% [21]. Suicidal ideation among physicians in two-volume meta-analyses reaches 17–17.4% [4, 22], while suicidal attempts reach 1.8% in lifetime [22]. These results are comparable to ours, and they are much higher than the general prevalence of suicidal ideation among the general population, which ranges through different investigations from 4.6 to 10.72% [23–25]. A high-volume meta-analysis pooled the prevalence of suicidal ideation for the general population at 12.1% [26]. A systematic review by García-Iglesias showed that during the COVID-19 pandemic, healthcare professionals reported increased suicidal ideation ranging from 2.4 to 21.7%, and 0.5–3.5% reported recent suicide attempts [27].

Secondly, we identified that relationship status, career change ideation, mobbing and exhaustion were associated with suicidality in our sample. Social isolation has strong links with suicide [28]. Additionally, the interpersonal theory of suicide suggests that thwarted belongingness, perceived burdensomeness [29] and loneliness [26] are associated with suicidal ideation. Career change ideation is a factor associated with burnout [30]. Burnout is prevalent among physicians [30–32]. If not addressed, burnout can be one of the factors increasing the risk of suicide [31]. Mobbing is a common risk factor in the workplace, even though it should be a strict taboo in any organisation. A study from Turkey evaluating mobbing experience by nurses found that 10% of participants were considering suicide [33]. Exhaustion was also proved to be a risk factor for suicidal risk in previous studies [26].

In our study, depression and anxiety were among the factors that had the largest prediction values for high suicide risk in the regression analysis in our sample. A meta-analysis published by Ribeiro et al. [34] found that depression and hopelessness were associated with a 1.96 increased risk for suicide ideation, 1.63 for suicide attempt, and 1.33 for death from suicide. However, the authors of this study admitted that some methodical constraints limited the expected effect of depression on suicidal behaviour. The Diagnostic and Statistical Manual of Mental Disorders 5th edition states that one of the main features of major depression is thoughts about death and suicide [35]. Furthermore, underlying anxiety can additionally increase suicide risk [36, 37]. A study by the World Health Organisation by Bertolote concluded that 98% of death by suicide had links with mental disorders and 30.2% with mood disorders [38]. Depression is associated heavily with physicians’ and residents’ lives. It starts in medical school, where it is shown that after medical school, depression symptoms increase by 13.5% [39]. Residents have a high prevalence of depression, which varies from 23.2 to 28% [40–42] and even 43% for frontline workers [41].

Regarding the limitations of this study, it is essential to note that study participants were self-referred and not randomly selected. This can lead to a selection bias, and results can show the state of the more active part of the medical doctor population in Lithuania, or those having higher mental health issues might have been more inclined to fill in the survey. Another limitation is that it is not a longitudinal study, so it is hard to determine how suicide risk changes over time. The study design did not allow us to evaluate the effects of the COVID-19 pandemic on the study findings. Our data collection was conducted in December 2021 and January 2022. At that time, Lithuania already dealt with two COVID-19 waves and successfully dealt with them [43]. In addition, vaccination was at the highest pace [44] and it was much more known how to deal with this disease. World Health Organisation prepared and extended guidelines [45] and personal protective equipment was available and accessible. The study data was collected when most medical doctors were vaccinated for COVID-19 in Lithuania. Lastly, the COVID-19 pandemic did not end, and the disease is a huge part of everyday doctors lives [46].

## Conclusions

This is the first empirical study to report suicidality in a large sample of the Lithuanian medical doctors and residents using the SBQ-R. The lifetime suicide risk was found in 30.4% of respondents. Moreover, 11.4% of respondents had suicidal planning ideation in their lifetime, and 2.5% reported previous suicide attempts. Multivariate binary regression showed that having no long-term relationships, higher depression and anxiety were significant suicide risk factors in medical doctors and residents. The results of the study can help to develop prevention strategies by identifying populations that may be at high risk of developing mental health symptoms and conditions and supporting experts in implementing specific interventions to address mental health problems and prevent suicide.

**Table 1:** Sociodemographic characteristics and suicide risk in Lithuanian medical doctors and residents

Variable	Overall population (*N* = 685)	SBQ-R* Score		*P*
		< 7 (low suicide risk) (*N* = 519)	≥ 7 (high suicide risk) (*N* = 208)	
Gender (%)				*P* = 0.279
Male	146 (21.3%)	107 (73.3%)	39 (26.7%)	
Female	539 (78.7%)	370 (68.6%)	169 (31.4%)	
Age (years, mean, SD)	39.57 (± 12.25)	40.63 (± 12.46)	37.14 (± 11.4)	*P* = 0.001
Relationships				
Not in a long-term relationship	129 (18.8%)	73 (56.6%)	56 (43.4%)	*P* < 0.001
In a long-term relationship	556 (81.2%)	404 (72.7%)	152 (27.3%)	
Having children				
Yes	389 (56.8%)	293 (75.3%)	96 (24.7%)	*P* < 0.001
No	296 (43.2%)	184 (62.2%)	112 (37.8%)	
Professions				
Medical doctor	561 (81.9%)	399 (71.1%)	162 (28.9%)	*P* = 0.072
Resident	124 (18.1%)	78 (62.9%)	46 (37.1%)	
Type of work				
Outpatient				
Yes	479 (69.9%)	337 (70.4%)	142 (29.6%)	*P* = 0.532
No	206 (30.1%)	140 (68.0%)	66 (29.6%)	
Inpatient				
Yes	343 (50.1%)	243 (71.1%)	99 (28.9%)	*P* = 0.42
No	342 (49.9%)	234 (68.2%)	109 (31.8%)	
Rehabilitation				
Yes	26 (3.8%)	22 (84.6%)	4 (15.4%)	*P* = 0.09
No	659 (96.2%)	455 (69.0%)	204 (31.0%)	
Nursing				
Yes	12 (1.8%)	11 (91.7%)	1 (8.3%)	*P* = 0.094
No	673 (98.2%)	466 (69.2%)	207 (30.8%)	
Emergency department				
Yes	188 (27.4%)	121 (64.4%)	67 (35.6%)	*P* = 0.065
No	497 (72.6%)	356 (71.6%)	141 (28.4%)	
Intensive care unit				
Yes	57 (8.3%)	36 (63.2%)	21 (36.8%)	*P* = 0.267
No	628 (91.7%)	441 (70.2%)	187 (29.8%)	
Level of medical service provision				
Primary				
Yes	285 (41.6%)	201 (70.5%)	84 (29.5%)	*P* = 0.669
No	400 (58.4%)	276 (69.0%)	124 (31.0%)	
Secondary				
Yes	323 (47.2%)	229 (70.9%)	94 (29.1%)	*P* = 0.497
No	362 (52.8%)	248 (68.5%)	114 (31.5%)	
Tertiary				
Yes	313 (45.7%)	221 (67.4%)	102 (32.6%)	*P* = 0.246
No	372 (54.3%)	266 (71.5%)	106 (28.5%)	
Primary workplace location				
One of the five biggest cities	542 (79.1%)	374 (69.0%)	168 (31.0%)	*P* = 0.776
Another smaller city	117 (17.1%)	84 (71.8%)	33 (28.2%)	
Township/rural area	26 (3.8%)	19 (73.1%)	7 (26.9%)	
Workload (Full-Time equivalent)				
< 1 FTE	72 (10.5%)	55 (76.4%)	17 (23.6%)	*P* = 0.418
1 FTE	236 (34.5%)	162 (68.6%)	74 (31.4%)	
> 1 FTE	377 (55%)	260 (69.0%)	117 (31.0%)	
Average work experience after finished training (years) *M* (*SD*)	14.75 (± 12.58)	15.72 (± 12.38)	12.38 (± 11.86)	*P* = 0.004
Had career change ideation in last 12 months				
Yes	471 (68.8%)	297 (63.1%)	174 (36.9%)	*P* < 0.001
No	214 (31.2%)	180 (84.1%)	34 (15.9%)	
Depression, according to DASS-21				
None	203 (29.6%)	178 (87.7%)	25 (12.3%)	*P* < 0.001
Mild	104 (15.2%)	82 (78.8%)	22 (21.2%)	
Moderate	192 (28%)	140 (72.9%)	52 (27.1%)	
Severe	91 (13.3%)	49 (53.8%)	42 (46.2%)	
Extremely severe	95 (13.9%)	28 (29.5%)	67 (70.5%)	
Anxiety, according to DASS-21				
None	253 (36.9%)	218 (86.2%)	35 (13.8%)	
Mild	69 (10.1%)	53 (76.8%)	16 (23.2%)	*P* < 0.001
Moderate	198 (28.9%)	133 (67.2%)	65 (32.8%)	
Severe	67 (9.8%)	33 (49.3%)	34 (50.7%)	
Extremely severe	98 (14.3%)	40 (40.8%)	58 (59.2%)	

* SBQ-R - The Suicidal Behaviors Questionnaire-Revised

**Table 2:** Work-related stress factors associated with high suicide risk in Lithuanian medical doctors and residents

Variable	Overall population	SBQ-R* Score		*P*
		< 7(low suicide risk)	≥ 7 (high suicide risk)	
Work-related stressors				
Poor working conditions				
Yes	282 (41.2%)	182 (64.5%)	100 (35.5%)	*P* = 0.015
No	403 (58.8%)	295 (73.2%)	108 (26.8%)	
High workload				
Yes	465 (67.9%)	313 (67.3%)	152 (32.7%)	*P* = 0.055
No	220 (32.1%)	164 (74.5%)	56 (25.5%)	
Direct contact with patients				
Yes	243 (35.5%)	142 (64.8%)	77 (35.2%)	*P* = 0.034
No	442 (64.5%)	335 (71.9%)	131 (28.1%)	
Lack of professional development				
Yes	219 (32%)	156 (71.2%)	63 (28.8%)	*P* = 0.061
No	466 (68%)	363 (77.9%)	103 (22.1%)	
Lack of career perspectives				
Yes	188 (27.4%)	120 (63.8%)	68 (36.2%)	*P* = 0.042
No	497 (72.6%)	357 (71.8%)	140 (28.2%)	
Managers				
Yes	222 (32.4%)	144 (64.9%)	78 (35.1%)	*P* = 0.06
No	463 (67.6%)	333 (71.9%)	130 (28.1%)	
Mobbing				
Yes	185 (27%)	109 (58.9%)	76 (41.1%)	*P* < 0.001
No	500 (73%)	368 (73.6%)	132 (26.4%)	
Exhaustion				
Yes	521 (76.1%)	345 (66.2%)	176 (33.8%)	*P* = 0.001
No	164 (23.9%)	132 (80.5%)	32 (19.5%)	

*SBQ-R - The Suicidal Behaviors Questionnaire-Revised

**Table 3:** Multivariate analysis of predictors for high suicide risk in Lithuanian physician and residents population (*N* = 685)

		Odds Ratio	95.0% C.I. for Odds Ratio		*P*
			Lower	Upper	
Step 1	Gender (male)	1.2	0.79	1.82	0.37
	Age	1.025	1.01	1.04	**0.001**
Step 2	Gender (male)	0.88	0.54	1.44	0.61
	Age	1.01	0.93	1.08	0.92
	Relationship status (no relationship)	2.71	1.63	4.48	**< 0.000**
	Having children	1.33	0.83	2.13	0.24
	Work experience	0.98	0.92	1.05	0.64
	Career change ideation	3.47	2.13	5.65	**< 0.000**
Step 3	Gender (male)	1.12	0.72	1.99	0.48
	Age	0.99	0.92	1.07	0.82
	Relationship status (no relationship)	2.71	1.62	4.53	**< 0.000**
	Having children	0.81	0.49	1.31	0.38
	Work experience	0.99	0.93	1.07	0.9
	Career change ideation	2.63	1.57	4.42	**< 0.000**
	Poor working conditions	1.11	0.73	1.7	0.63
	Direct contact with patients	1.22	0.81	1.85	0.33
	Lack of career perspectives	1.18	0.75	1.86	0.46
	Mobbing	1.66	1.04	2.63	**0.03**
	Exhaustion	1.56	0.93	2.62	0.08
Step 4	Gender (male)	0.77	0.45	1.34	0.36
	Age	0.99	0.91	1.08	0.87
	Relationship status (no relationship)	2.48	1.42	4.32	**0.001**
	Having children	0.92	0.55	1.55	0.75
	Career change ideation	1.58	0.91	2.76	0.1
	Poor working conditions	1.01	0.64	1.58	0.98
	Direct contact with patients	1.11	0.71	1.71	0.67
	Lack of career perspectives	0.93	0.57	1.53	0.78
	Mobbing	1.14	0.69	1.89	0.61
	Exhaustion	1.19	0.68	2.08	0.54
	Depression				
	Normal	1	0.68	3.13	0.33
	Mild	1.46	0.91	3.49	0.09
	Moderate	1.77	1.48	7.66	**0.004**
	Severe	3.38	3.01	16.67	**< 0.001**
	Extremely severe	7.09			
	Anxiety				
	Normal	1			
	Mild	0.91	0.39	2.1	0.82
	Moderate	1.68	0.93	3.04	0.083
	Severe	2.94	1.32	6.56	**0.008**
	Extremely severe	2.19	1.01	4.73	**0.047**

*Note* CI – confidence interval

## Data Availability

No datasets were generated or analysed during the current study.
